# Proton pencil beam scanning lattice radiotherapy: technical implementation and comparison against photon-based delivery

**DOI:** 10.1093/bjr/tqag125

**Published:** 2026-05-23

**Authors:** Abdul S Khalid, Adam H Aitkenhead, Matthew Lowe, Matthew Clarke, John-William Warmenhoven, Francesca Fiorini, Thomas W S Satherley, Maxwell Robinson, Geoff S Higgins, Kristoffer Petersson

**Affiliations:** Christie Medical Physics and Engineering, The Christie NHS Foundation Trust, Manchester Academic Health Science Centre (MAHSC), Manchester M20 4BX, United Kingdom; Christie Medical Physics and Engineering, The Christie NHS Foundation Trust, Manchester Academic Health Science Centre (MAHSC), Manchester M20 4BX, United Kingdom; Division of Cancer Sciences, Faculty of Biology Medicine and Health, The University of Manchester, Manchester M13 9PL, United Kingdom; Christie Medical Physics and Engineering, The Christie NHS Foundation Trust, Manchester Academic Health Science Centre (MAHSC), Manchester M20 4BX, United Kingdom; Division of Cancer Sciences, Faculty of Biology Medicine and Health, The University of Manchester, Manchester M13 9PL, United Kingdom; Christie Medical Physics and Engineering, The Christie NHS Foundation Trust, Manchester Academic Health Science Centre (MAHSC), Manchester M20 4BX, United Kingdom; Division of Cancer Sciences, Faculty of Biology Medicine and Health, The University of Manchester, Manchester M13 9PL, United Kingdom; Christie Medical Physics and Engineering, The Christie NHS Foundation Trust, Manchester Academic Health Science Centre (MAHSC), Manchester M20 4BX, United Kingdom; Division of Cancer Sciences, Faculty of Biology Medicine and Health, The University of Manchester, Manchester M13 9PL, United Kingdom; Department of Radiotherapy Physics, Churchill Hospital, Oxford University Hospitals NHS Trust, Oxford OX3 7LE, United Kingdom; Department of Radiotherapy Physics, Churchill Hospital, Oxford University Hospitals NHS Trust, Oxford OX3 7LE, United Kingdom; Department of Radiotherapy Physics, Churchill Hospital, Oxford University Hospitals NHS Trust, Oxford OX3 7LE, United Kingdom; Department of Oncology, University of Oxford, Oxford OX3 7DQ, United Kingdom; Department of Oncology, University of Oxford, Oxford OX3 7DQ, United Kingdom

**Keywords:** lattice radiotherapy, SFRT, proton therapy, VMAT

## Abstract

**Objectives:**

Spatially fractionated radiotherapy in the form of a lattice of high-dose regions within the tumor has potential to improve the therapeutic response compared to standard radiotherapy. We aimed (1) to develop and dosimetrically validate a proton pencil-beam scanning lattice radiotherapy (LRT) planning technique, (2) to compare against a photon VMAT LRT technique, and (3) to assess which clinical sites are feasible for treatment.

**Methods:**

10 cases were selected, covering sites in the brain, neck, pelvis, abdomen and shoulder with gross tumor volumes greater than 100 cm^3^. LRT plans were developed for a Varian ProBeam system, aiming to deliver 20 Gy to the lattice vertices while minimizing dose to surrounding tissues. Plan-specific quality assurance (PSQA) was performed by independent Monte-Carlo calculation and physical dosimetric verification. End-to-end tests were performed using a CIRS head phantom containing EBT4 film. Plan quality was evaluated against VMAT LRT.

**Results:**

Clinically acceptable proton LRT plans were produced, delivering 16-20 Gy to the lattice vertices, a mean PTV dose of 2 Gy and satisfying organ-at-risk dose constraints. PSQA and end-to-end tests met clinical tolerances for non-LRT plans. For large target volumes, proton LRT produced lattices with a greater number of vertices than VMAT LRT.

**Conclusions:**

Proton LRT is technically viable using currently available delivery systems. Large tumors such as sarcoma are the most likely candidates for future clinical studies, but proton LRT is feasible for target volumes as small as 100 cm^3^.

**Advances in knowledge:**

The study demonstrates the feasibility of proton LRT for early phase clinical trials.

## Introduction

Spatially fractionated radiotherapy (SFRT) encompasses a number of related techniques.^1^ In clinical applications spatial fractionation was first used in the early 1900s in the form of Grid therapy^2^ making use of a collimator to produce beamlets of 1-2 cm in width. This results in a dose distribution that is a spatially fractionated grid in 2-dimensions. Originally, it was primarily used to spare normal tissues (such as the skin) during treatment of deep-seated tumors using orthovoltage X-rays. It became less commonly used following the introduction of megavoltage photon beams, which inherently gave better skin sparing.

In recent decades, interest in spatial fractionation has grown since it has been found to have benefits in terms of tumor control, especially for large or radioresistant tumors,^3^ and may play a role in priming an immune response for subsequent therapies.^4^^,^^5^ In the early 2000s the 2D Grid approach was extended to 3-dimensions in the form of lattice radiotherapy (LRT).^6^ In LRT, high doses are located on a 3D set of vertices within the overall target volume. Various technologies can be used to deliver the radiation, including multi-leaf collimated linear accelerators (either delivering fixed-field IMRT or dynamic arcs), a linear accelerator on a robotic arm, or pencil-beam scanning (PBS) ion beams.^6^ Since then, most clinical implementations of LRT have used linear accelerator-based technologies.

A wide variety of clinical sites have been treated using photon-based Grid therapy or LRT.^7–17^ Planning and feasibility studies for volumetric modulated arc therapy (VMAT)-based LRT have also been reported for sites including pelvis, abdomen, mediastinum, lung and neck^18^; lung, abdomen, pelvis and extremities^19^ and for brain and a range of sarcoma sites.^20^

Proton SFRT is not as widely implemented as photon SFRT, but has also been reported for a range of clinical sites. A treatment planning feasibility study of proton PBS Grid therapy showed that spatial fractionation could be produced within normal tissues in the entrance path of proton beams while maintaining a homogeneous dose to deep-seated target volumes.^21^ The clinical use of proton Grid therapy for sites including base-of-skull, head and neck, abdomen and pelvis has also been reported,^22^ demonstrating spatial fractionation within the target volume. They also reported that for some challenging anatomies proton SFRT may be feasible where the proximity of organs-at-risk (OARs) makes photon SFRT infeasible. A planning study comparing proton PBS Grid therapy against megavoltage photon Grid and Lattice techniques has also been reported.^23^

SFRT studies using current clinical proton PBS systems have focused mainly on Grid therapy rather than LRT, although several novel techniques have been reported including LRT via arc therapy^24^ and minibeam SFRT via collimated beams.^25^ For the present study, we investigated the implementation of proton LRT using existing clinical approaches and equipment for delivery of PBS proton therapy in the United Kingdom.

This study was intended to be a first step toward enabling early phase clinical proton LRT trials in the United Kingdom. Therefore, we aimed (1) to develop a planning approach for proton LRT that is generalizable for a range of anatomical sites and perform measurements to ensure that generated plans are deliverable, (2) to dosimetrically evaluate the proton LRT plans in comparison to a VMAT LRT technique, (3) to assess which clinical sites are potentially feasible for treatment by proton LRT.

## Methods

### Cohort details

This retrospective study was conducted in collaboration between the Christie NHS Foundation Trust and Oxford University Hospitals NHS Trust. A cohort of 10 cases was drawn from patients treated using proton beam therapy (PBT) at the Christie and VMAT at Oxford as follows.

We defined a lattice as a set of high-dose vertices within the gross tumor volume (GTV), each 1 cm in diameter, with a 3-4 cm center-to-center spacing and a minimum gap of 0.5 cm to the edge of the GTV. Based on this geometry, the GTV volume must be at least 100 cm^3^ to allow a minimum of 3 vertices to be defined within the lattice. Using this as a selection criterion, a retrospective audit of all 520 patients treated using PBT at the Christie between 1 January 2022 and 31 December 2023 was performed to identify potential cases for the study.

The majority of PBT treatments at the Christie are pediatric cases due to the reduction in dose to normal tissues that can be achieved using proton rather than megavoltage photon beams. However, GTV volumes for most pediatric cases did not meet the minimum volume criterion. The next most treated sites were central nervous system (CNS) and base-of-skull tumors. Whole craniospinal irradiation and bone cases were considered unsuitable for early phase LRT studies and were excluded from the outset. Treatment sites within the brain that met the minimum GTV criterion were further screened to ensure critical structures did not have significant overlap with the GTV.

Five suitable PBT cases were selected for LRT planning: 3 sarcomas (2 soft-tissue sarcomas spanning the neck to the patient midline and 1 pelvic chondrosarcoma) and 2 brain cases (1 low-grade glioma and 1 CNS parietal lobe tumor). A further 5 cases were selected from patients previously treated using VMAT at Oxford, all soft-tissue sarcomas in the pelvis, abdomen and shoulder. All 10 cases were anonymized prior to planning with proton and VMAT LRT.

### Proton LRT planning implementation

**Figure 1:**
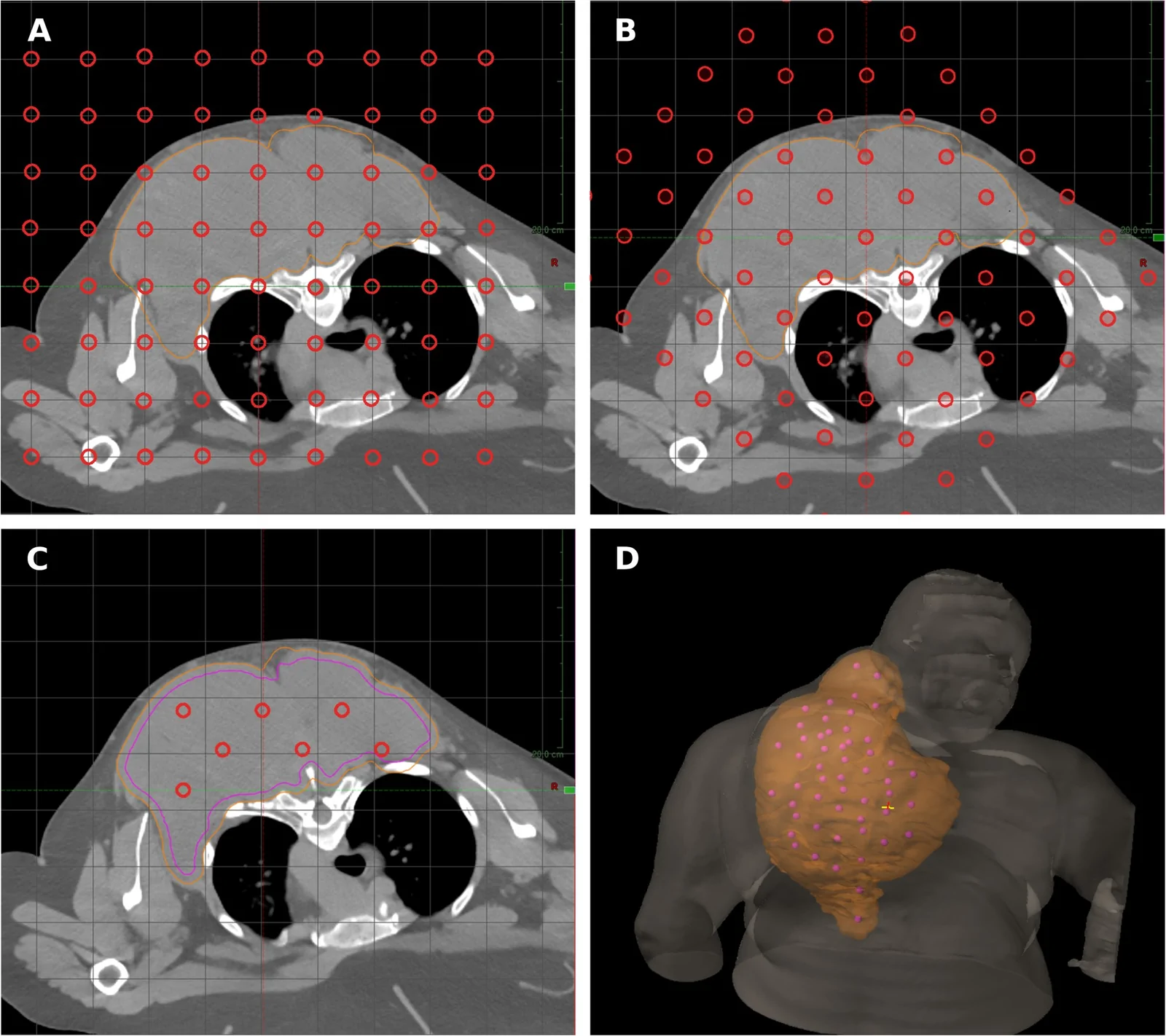
Illustration of the stages of vertex placement for a sarcoma case (GTV = 5300 cm^3^), showing (A) the initial grid of spheres extended throughout the patient volume, (B) the grid rotated through 45°, and (C) the grid cropped to the GTV minus 0.5 cm contraction. (D) A 3D render of the final grid of 52 vertices. Orange = GTV; Blue = GTV - 0.5 cm; Magenta = high-dose vertices.

A proton LRT planning technique was developed using the Varian (Palo Alto, CA, United States) Eclipse v18 treatment planning system (TPS) and Varian ProBeam PBS delivery system. A methodology was determined for placement of high-dose vertices within the GTV that would result in the desired peak-to-valley dose distribution of a typical LRT plan. The Eclipse grid tool was used to create a set of 1 cm diameter spheres on a 3D Cartesian grid with a lattice spacing of 3-4 cm. This grid was extended throughout the patient, then rotated 45° in the transverse plane. The rotation improved the dose separation between high-dose islands in the final plans. Vertices were removed outside the GTV minus 0.5 cm contraction, and from within a 1 cm expansion of OARs including skin. The vertex placement method is demonstrated for a sarcoma case in Figure 1.

In addition to the relevant OARs, the following LRT-specific structures were delineated for use in plan optimization and dosimetric analyses. These are based on the VMAT LRT approach described by Ref.,^20^ with adjustments made to aid proton LRT plan optimization:

- vertex#: Each individual high-dose vertex, defined as a 1 cm diameter sphere.
- vertices: All 1 cm diameter spheres grouped together.
- CTV: The clinical target volume (CTV), contoured by the clinician.
- PTV: The planning target volume (PTV), defined as a 1 cm expansion of the CTV.
- PTV-vertices: A Boolean subtraction of all vertices from the PTV.
- GTV-(vertices+1.0cm): A Boolean subtraction of all vertices (plus 1.0 cm expansion) from the GTV. The first of a series of concentric rind-like structures within GTV.
- GTV-(vertices+1.5cm): A Boolean subtraction of all vertices (plus 1.5 cm expansion) from the GTV.
- GTV-(vertices+2.0cm): A Boolean subtraction of all vertices (plus 2.0 cm expansion) from the GTV.
- Rind_inner: A 1 cm rind outside of the CTV used to control the dose fall-off outside of CTV.
- Rind_outer: A 2 cm rind outside of Rind_inner to further control the dose fall-off outside of CTV.
- Body-PTV: The patient body contour with the PTV removed to control dose outside of the PTV.

**Figure 2:**
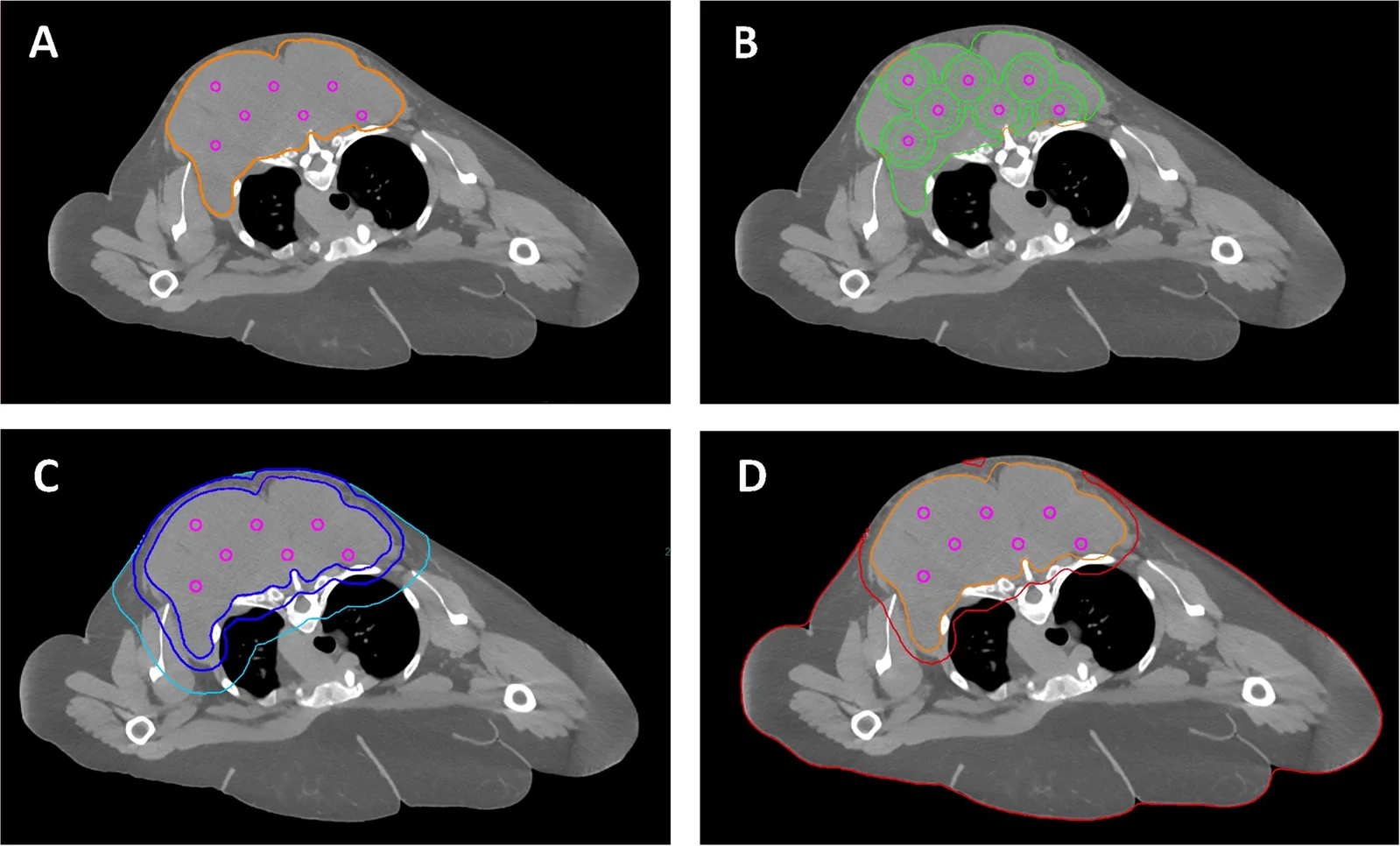
Illustration of the key optimization structures for a sarcoma case (GTV = 5300 cm^3^), showing: (A) GTV (orange) and spheres (magenta), (B) GTV-(sph+1.0 cm) (dotted green), GTV-(sph+1.5 cm) (dashed green), GTV-(sph+2.0 cm) (solid green), (C) Rind_inner (blue), and Rind_outer (cyan), (D) Body-PTV (red).

These structures are demonstrated in Figure 2 for the same sarcoma case shown in Figure 1. In this example, GTV and CTV were identical. The concentric expansions of the vertices subtracted from the GTV were used to guide the optimizer to smoothly reduce dose from the high-dose vertices.

Treatment planning was carried out to meet the plan objectives shown in Table 1, which are based on those described by Ref.^20^ Optimal and mandatory objectives as well as priority levels were defined for each metric. The priorities were:

**Table 1:** The planning objectives which were used for optimization of all proton LRT and VMAT LRT plans.

Volume	Metric	Optimal	Mandatory	Priority
Body—PTV	*D*_1cc_	<2 Gy	<4 Gy	1
Each OAR	*D*_0.5 cc_	<2 Gy	<3 Gy	1
Large parallel OARs^a^	*D*_mean_	<2 Gy	<3 Gy	1
Each vertex	*D*_95%_	20 Gy	>16 Gy	2
All vertices	*D*_mean_	20 Gy	>16 Gy	2
All vertices	*D*_0.1 cc_	<140% of prescribed dose^b^	<160% of prescribed dose^b^	2
GTV	*D*_0.5%_	>20 Gy	>16 Gy	2
PTV—vertices	*D*_mean_	<2 Gy	<3 Gy	3
GTV—(vertices + 2.0 cm)	*D*_0.1 cc_	<4-6 Gy		3
PTV	Conformity index for 2 Gy: Vol(2 Gy in Body)/Vol(2 Gy in PTV)	<1.15		3

Objectives had 2 levels: “Optimal,” representing the ideal value; “Mandatory,” representing a lower required value. Objectives are based on those described by Ref.^20^

a For example, lung, liver, kidneys, skin.

b For proton plans: 20 Gy (*D*_mean_) to vertices. For VMAT plans: 16-20 Gy (*D*_0.5%_) to GTV.

1. To control the dose to OARs and normal tissues to minimize the risk of inducing significant treatment toxicity.
2. To control the dose to the vertices to maximize the treatment efficacy of LRT.
3. To control the dose to the PTV, ensuring that the dose falls off tightly around the vertices while keeping the mean dose to the PTV in the region of 2 Gy; and to ensure conformity of the dose to the PTV.

Fields were spaced at 45° intervals and aligned with the lattice to ensure beam paths intersected multiple vertices, such that both the peak and entrance (plateau) sections of beams contribute dose to lattice vertices.

The number of fields was kept as low as possible. However, the use of too few beams led to high doses in the beam entrance region, so keeping the skin dose within an acceptable range was the main driver for the choice of the number of fields. Fields were chosen to enter on the same side as the treatment site wherever possible to minimize contralateral dose. Midline and central target volumes used fields spread out around the patient. As per standard PBT approaches, field angles were chosen to ensure beams did not enter through critical OARs or regions and directions of motion uncertainty. Multi-field optimization was used for all plans. The planning approach meant that range shifters were not required, allowing smaller proton spot sizes and sharper lateral dose fall-off. Robust optimization was not applied. Plans were prescribed in terms of the mean dose (20 Gy) to the vertices.

The overall planning approach was similar to current clinical planning for standard intensity modulated proton therapy (IMPT). Energy layer spacing was energy dependent, ranging from 2.1 MeV spacing at 70 MeV to 2.8 MeV at 200 MeV. None of the LRT plans required energies greater than 200 MeV. Pencil-beam spacing was 1.3σ_E_ where σ_E_ is the Gaussian width in air at isocentre of a single pencil-beam of energy E. The final lattice plans were within the typical range of clinical plans in terms of monitor units and the relative contribution of dose per field.

### Dosimetric comparison: proton LRT vs VMAT LRT

VMAT LRT plans were planned according to the approach described by Ref.,^20^ using a Varian Eclipse v16 TPS with a 10 MV flattening filter-free (FFF) Truebeam beammodel for the sarcoma cases, and 6 MV FFF for the brain cases. The VMAT and proton LRT approaches were developed in parallel and shared the same set of planning objectives (Table 1) and GTV, CTV, PTV, and OAR structures. Differing from the proton LRT approach, for VMAT LRT the prescription specified the near-maximum dose (*D*_0.5%_) to the GTV and was set to a value of 20 Gy, although this could be relaxed to 16 Gy if necessary to meet the priority 1 objectives in Table 1. Additionally, in the VMAT approach having more than 3 vertices in any axial plane was not feasible due to need to avoid undeliverable multi-leaf collimator segments. Consequently, for large GTVs the number of vertices in the VMAT solution was reduced if necessary. If the number of vertices was reduced by more than half, the diameter of the vertices was increased to compensate for the reduction in lattice volume. This was done for 2 VMAT plans: Shoulder-3 (vertex diameter 1.5 cm) and Shoulder-4 (diameter 2.0 cm).

During planning, it was mandatory for all plans to meet the standard OAR and normal tissue tolerances for the respective clinical sites. The final plans were compared using additional metrics to assess the dose to the vertices (both individually and as a complete set), the dose to the target (GTV, CTV and PTV) and the dose to the target excluding the vertices. The dose to skin was evaluated within a 5 mm rind of the patient external contour.

Note that for ease of comparison, within the text, table and figures the units of dose for both proton and VMAT LRT plans are stated as Gy. For proton plans, the units should formally be Gy_RBE=1.1_ since a proton relative biological effect (RBE) of 1.1 was implemented within the TPS proton beam-model.

### Deliverability: plan-specific quality assurance for proton LRT

Plan-specific quality assurance (PSQA) was performed for all plans to validate the accuracy of the TPS dose calculation and the deliverability on a Varian ProBeam system. Within the TPS, dose calculations were performed using an analytical proton-convolution-superposition (PCS) algorithm.^26^ All plans were independently re-calculated using an in-house Monte-Carlo (MC) system^27^ based on Gate v.8.1^28^ and Geant4 v.10.3.3.^29^ This enabled the dose calculation within the patient CT to be validated.

Deliverability was validated via physical dosimetry. For each plan, each field was delivered with the gantry at 0° (ie, with the beam aimed vertically downwards) to a stack of solid-water containing a PTW 1500XDR 2D array and Gafchromic film (either EBT3 or EBT4). The array was positioned at a suitable depth for each field, dependent on the energy of the layers in the field. The film was located within the solid-water stack 1 cm upstream of the array.

All films were scanned using an Epson 12000XL flatbed scanner in transmission RGB mode at a spatial resolution of 200 dpi and at 16-bits per color channel, with the resulting images stored in TIFF format. Films were scanned at least 24 hours after irradiation.^30^ A correction was applied to all images to account for the non-uniform lateral response of the scanner, using a method similar to Lewis and Chan.^31^ A set of film calibration images was irradiated at doses from 0 to 40 Gy. Calibration curves were generated for each RGB channel for doses from 0 to 10 Gy, with the higher dose films used to check for saturation of the films. PSQA was done on a per-field basis, so the maximum dose delivered per field was lower than 20 Gy (eg, for a 4 field plan the maximum dose per field was closer to 5 Gy).

All PSQA approaches (MC, film and array) were compared to TPS dose calculations using gamma analysis,^32^ where the MC, film or array data was the reference dataset and the TPS was the evaluated (ie, interpolated) dataset. It should be noted that the reference dataset was normalized to the evaluation dataset prior to the gamma analysis, so that the analysis was a test of the relative dose distribution only. The magnitude of the delivered dose was assessed separately, by evaluating the normalization factors derived during the analysis of the MC and film data.

### Deliverability: end-to-end testing

End-to-end testing was performed using a CIRS proton head phantom.^33^ Two LRT plans were created to treat a hypothetical target in the right hemisphere of the brain where the film planes were located, using 6 and 10 high-dose vertices, including vertices centered on one of the film planes. Both plans used 3 fields, with field angles chosen to avoid the beam being aimed along the plane of the films. No range-shifter was used for either plan. The plans were delivered to the phantom, with Gafchromic film used to measure the delivered dose for comparison against the TPS and independent MC dose calculations. For the end-to-end tests, film calibration curves were generated up to 25 Gy to cover the full range of delivered doses.

## Results

### Dosimetric comparison: proton LRT vs VMAT LRT

**Figure 3:**
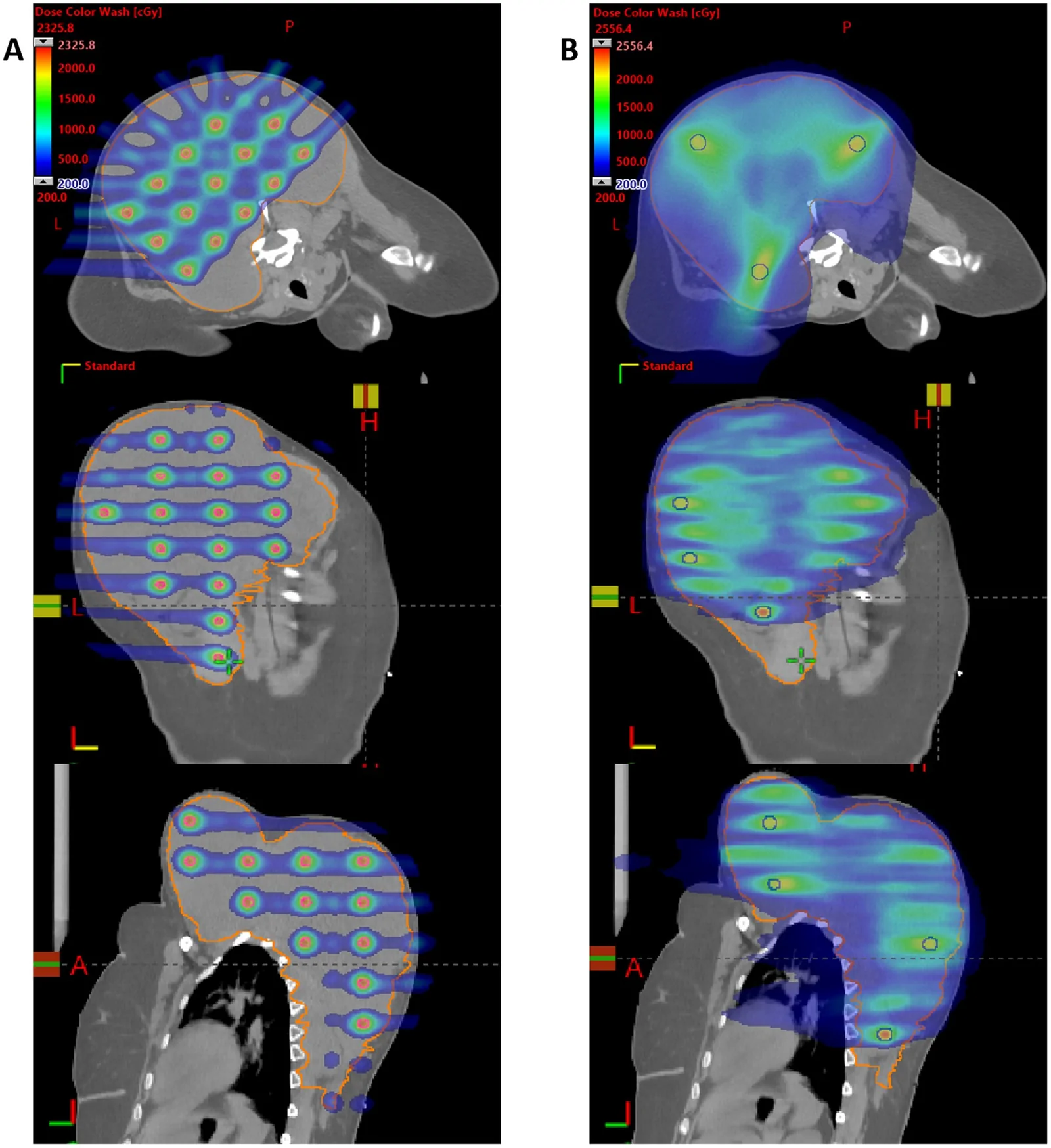
The TPS dose distributions for a sarcoma case (GTV = 5300 cm^3^) planned using (A) proton LRT and (B) VMAT LRT techniques. The plans contained 52 (for protons) and 20 (for VMAT) high-dose vertices.

Example proton and VMAT LRT plans for a sarcoma case and a brain case are shown in Figures 3 and Figure SA1, respectively.

**Figure 4:**
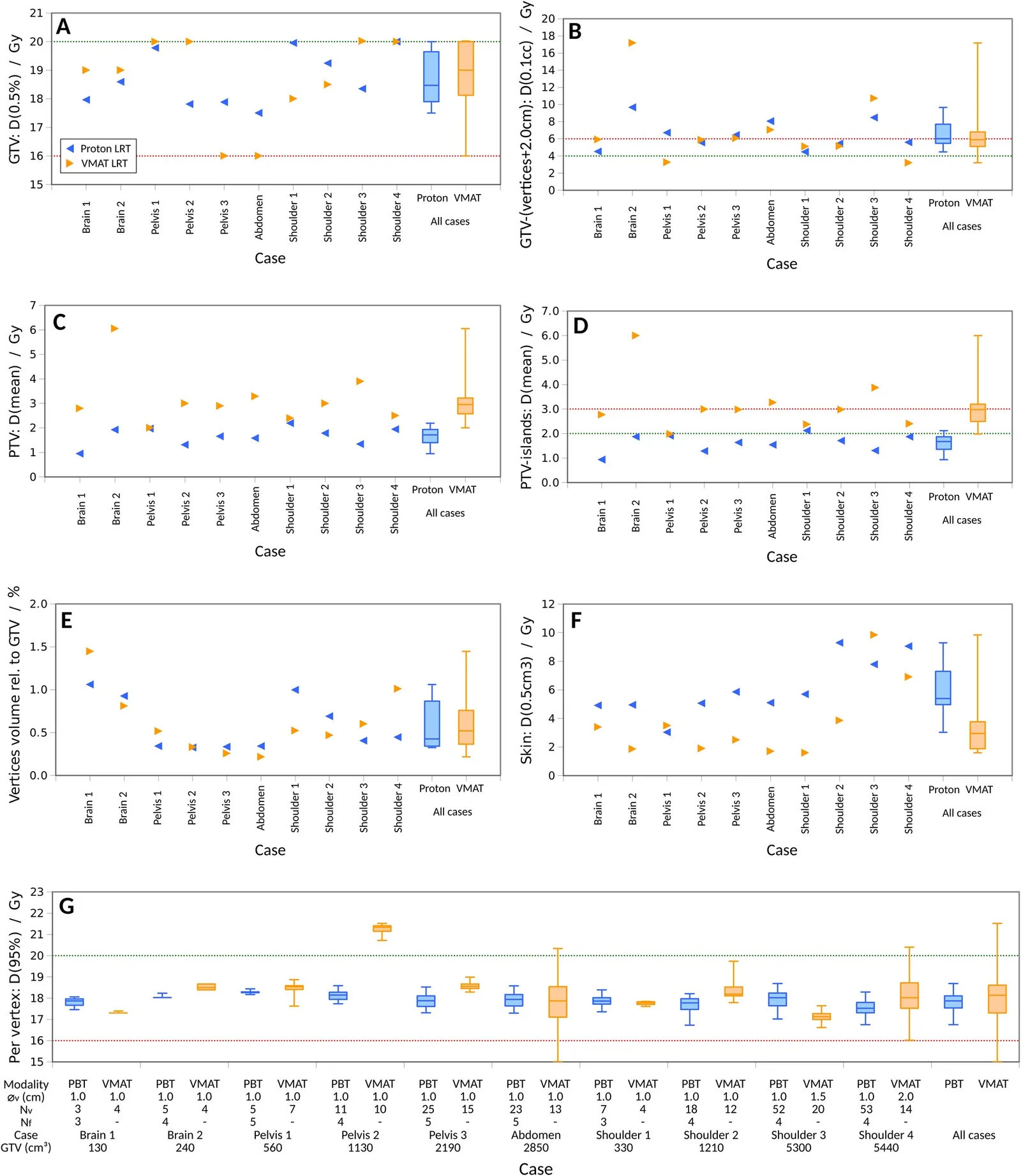
Summary of the proton LRT and VMAT LRT plan metrics for all 10 cases. (A) *D*_0.5%_ to the GTV. (B) $D_{\text{max}0.1\;{\text{cm}}^{\text{3}}}^{}$ to the GTV minus the vertices including a 2 cm margin. (C) *D*_mean_ to the PTV. (D) *D*_mean_ to the PTV minus the high-dose vertices. (E) The volume of the vertices relative to the GTV volume. (F) $D_{0.5\;{\text{cm}}^{3}}^{}$ to the skin. (G) Box plots summarizing the distributions of *D*_95%_ values for all vertices in each plan, showing the median (center line), inter-quartile range (box) and range (whiskers). Optimization objectives (taken from Table 1) are marked where applicable: green dotted line = optimal; red dotted line = mandatory. Abbreviations: Ø_v_ = Diameter of the vertices; N_v_ = Number of vertices; N_f_ = Number of fields.

Across all 10 cases the variation of the near-maximum dose to the GTV (*D*_0.5%_) varied between 16-20 Gy for all proton and VMAT LRT plans (Figure 4A), showing good consistency despite the differing prescription approaches.

Several proton LRT plans exceeded the 6 Gy limit for the near-maximum dose (*D*_0.1 cc_) to the GTV (excluding the high-dose vertices, GTV-vertices + 2.0 cm). However, this metric was given a low priority during optimization, and its purpose was to avoid high doses being placed outside lattice vertices (Figure 4B). It was not intended to be used to evaluate plan quality.

Values for the mean dose to the PTV and to PTV excluding vertices (PTV-vertices) were similar due to the small volume of the vertices relative to the PTV (Figure 4C and D). The volume of all vertices was never greater than 1.5% of the GTV volume (Figure 4E). The proton plans were more readily able to meet the optimal mean PTV dose of 2 Gy, while VMAT plans were typically close to the mandatory objective of 3 Gy.

The near-maximum dose to the skin (*D*_0.5 cc_) ranged from 3.0 to 9.3 Gy for proton LRT, and 1.6-9.9 Gy for VMAT LRT (Figure 4F). Proton plans generally resulted in a higher skin dose (mean 6.1 Gy) than VMAT (mean 3.7 Gy), a consequence of the lack of a skin-sparing effect for proton beams. Although higher than VMAT, all proton LRT skin doses were within the typical range of standard clinical proton treatments.

All proton plans lay within the 16 Gy objective for dose covering 95% of the volume for each vertex (Figure 4G). One of the VMAT plans (“Abdomen”) contained vertices that lay below the 16 Gy objective, but since the majority were above 16 Gy it is likely that the plan would still be clinically suitable. For VMAT plan “Pelvis 2,” the mean *D*_95%_ per vertex was 21.3 Gy, above the target of 20 Gy. However, the plan met all other objectives, and if desired could easily be re-weighted to fine-tune the dose to the vertices.

### Deliverability: plan-specific quality assurance

**Figure 5:**
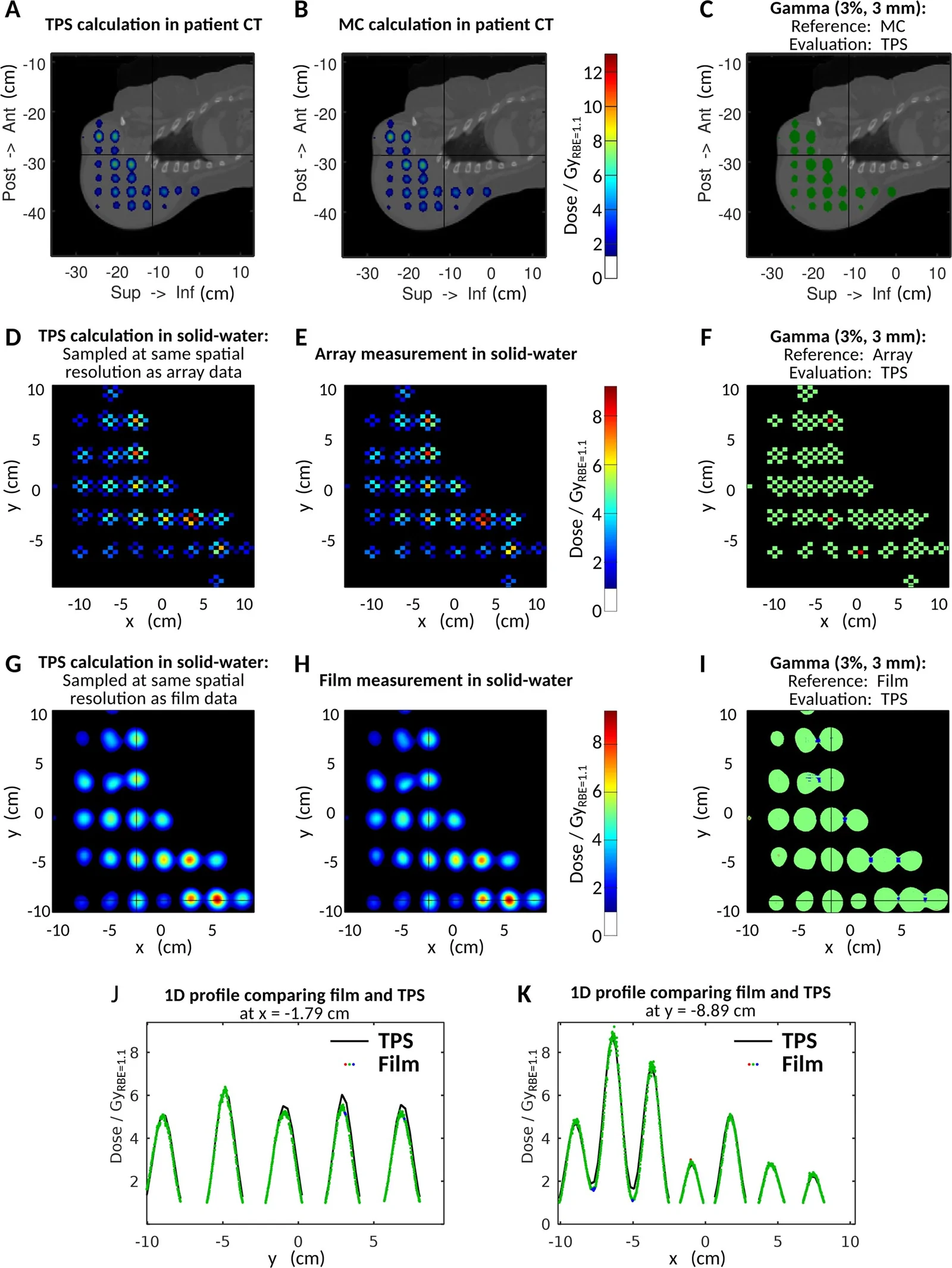
PSQA results for one field of the proton LRT plan for the sarcoma case shown in Figures 1-3. (A-C) A sagittal plane of the patient CT image overlaid by the TPS dose calculation, the Monte-Carlo dose calculation and the results of a gamma analysis comparing TPS and MC for gamma criteria 3% (relative to local dose) and 3 mm, with doses <10% of the maximum excluded from analysis. (D-F) TPS, array and gamma [3% (relative to local dose), 3 mm, with doses <10% of the maximum excluded from analysis]. (G-I) TPS, film and gamma (3% (relative to local dose), 3 mm, with doses <10% of the maximum excluded from analysis). (J, K) TPS and film 1D profiles. In panels C, F, I, J, K the gamma results are colour-coded as follows: Green: γ ≤ 1; Blue: γ > 1 and Reference < Evaluation dataset; Red: γ > 1 and Reference > Evaluation dataset.

Example proton LRT PSQA results for the sarcoma case introduced in Figures 1-3 are shown in Figure 5. MC dose calculations verified that the TPS calculations in patient CT were in good agreement with independent MC results (Figure 5A-C). Measurements using an array (Figure 5D-F) and GafChromic EBT3 film (Figure 5G-I) in a solid-water phantom verified the plan delivery in comparison to the TPS dose calculation. Analysis of 1D profiles through the vertices for film and TPS verified the dose to the peaks and valleys of the lattice. Note that the peak doses shown in Figure 5 are less than the prescribed dose of 20 Gy since PSQA was done on a per-field basis.

**Figure 6:**
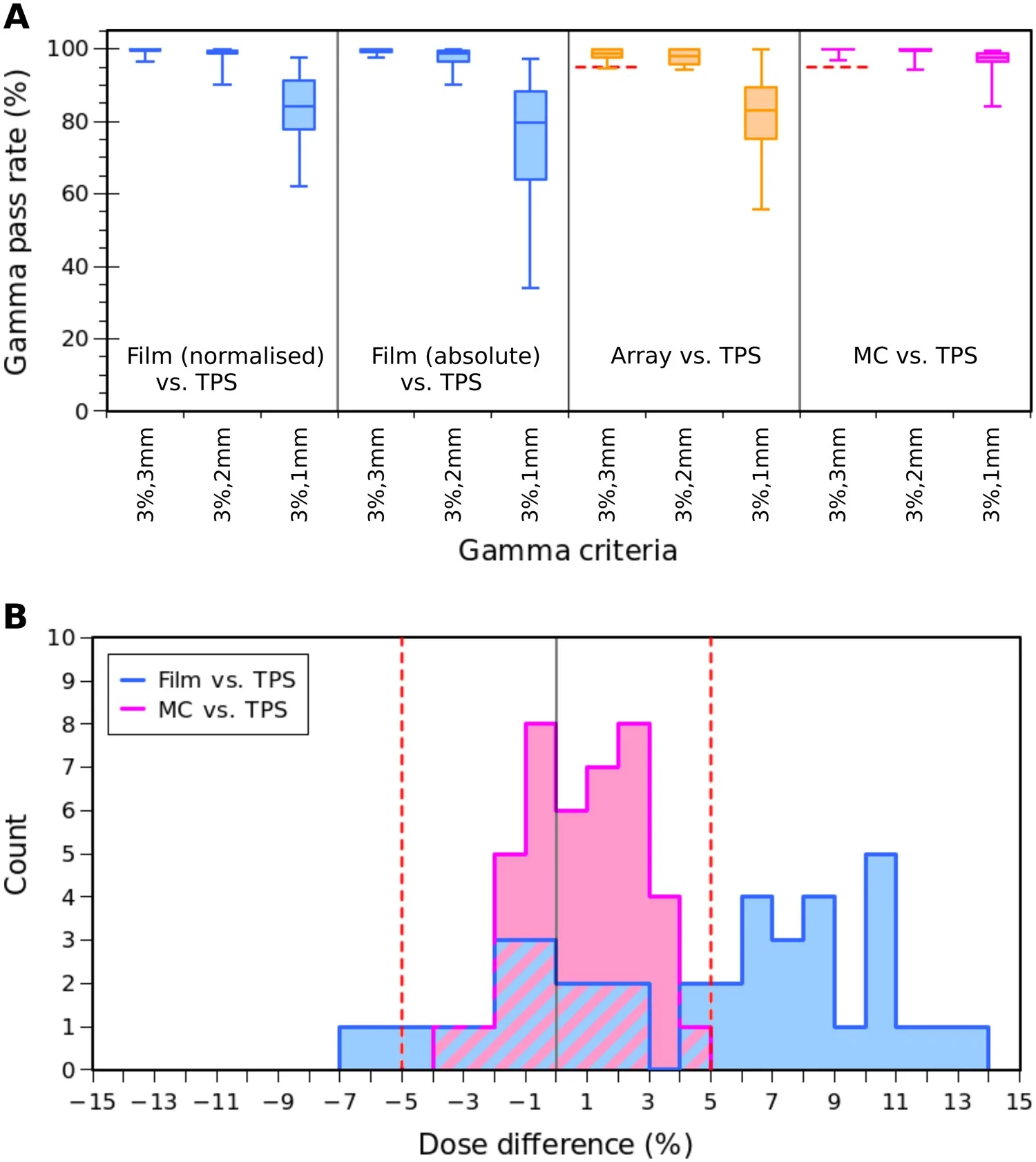
Summary of proton LRT PSQA results for all 10 cases (41 fields). (A) Summary of gamma analysis results, comparing Film, Array and MC vs TPS. Doses were evaluated relative to the local dose. Data below 10% of the maximum dose was excluded. The film/array/MC datasets were taken as the reference datasets, and the TPS was the evaluation dataset. The red dashed lines indicate the current clinical tolerance for array and MC verifications of non-SFRT plans. The box-plots show the median (centre line), inter-quartile range (box) and range (whiskers). (B) Dose difference between MC or film and TPS. Dose differences were quantified by the normalization factor determined prior to the gamma analysis. Positive values indicate that the TPS dose > MC or film. The red dashed lines indicate the current clinical tolerance for MC verifications of non-SFRT plans.

Gamma analyses for all 10 cases (41 fields) with MC, array and film lay within clinical tolerances for standard IMPT plans, with >95% of points having γ ≤ 1 at 3%, 3 mm for each field (Figure 6A). The magnitude of the delivered dose was assessed using the normalization factors derived from the MC and film datasets in comparison to the TPS (Figure 6B). The MC dose calculations were found to be systematically 0.9 ± 1.9% (mean ± 1 standard deviation) colder than the TPS, which was within the clinical tolerance (of ±5%) for standard IMPT plans. Film measurements were 4.3 ± 5.4% colder than the TPS. We do not use film for clinical PSQA of standard IMPT plans, so do not have a defined clinical tolerance. Evaluating the dose delivered to localized vertices to high accuracy is challenging, but these results demonstrate that both MC and film results are consistent with the TPS dose calculation, verifying that the plans do not have any gross calculation or delivery errors. In combination with routine machine QC, the MC simulations and dosimetric measurements demonstrate that proton LRT plans can be delivered accurately.

### Deliverability: end-to-end testing

**Figure 7:**
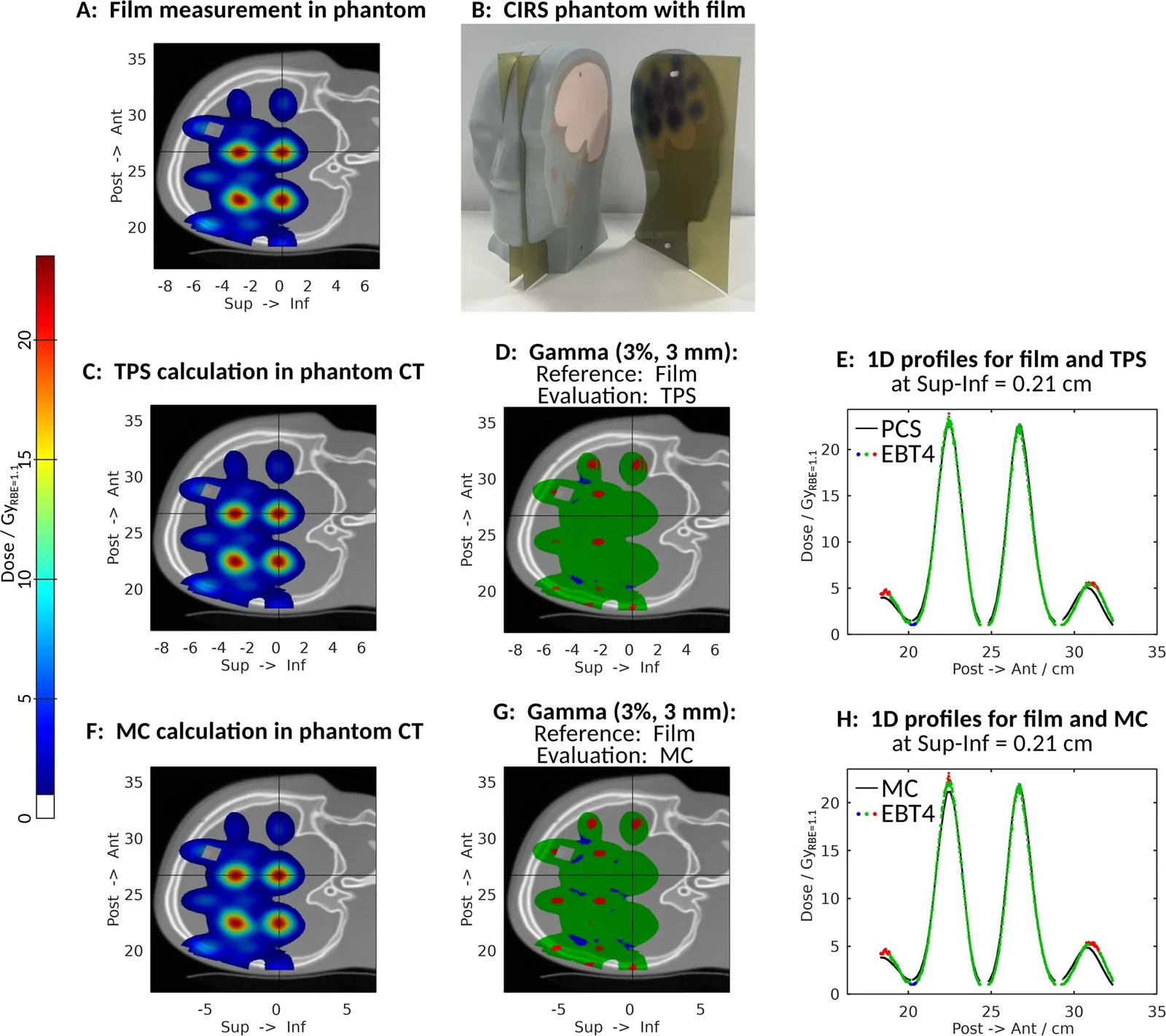
End-to-end test for a 10-vertex proton LRT plan delivered to a CIRS head phantom. (A) EBT4 film measurement of the dose delivered to (B) the CIRS head phantom (Photo: Lilia Hill). (C) The TPS dose calculation. (D) Gamma analysis comparing film and TPS. (E) 1D dose profile in the anterior-posterior direction comparing film and TPS. (F) The MC dose calculation. (G) Gamma analysis comparing film and MC. (H) 1D dose profile in the anterior-posterior direction comparing film and MC. Results in (D, E, G, H) used gamma settings of 3% (relative to local dose), 3 mm, with doses <10% of the maximum excluded from analysis, and are colour-coded as follows: Green: γ ≤ 1; Blue: γ > 1 and EBT4 < Evaluation dataset; Red: γ > 1 and EBT4 > Evaluation dataset.

**Figure 8:**
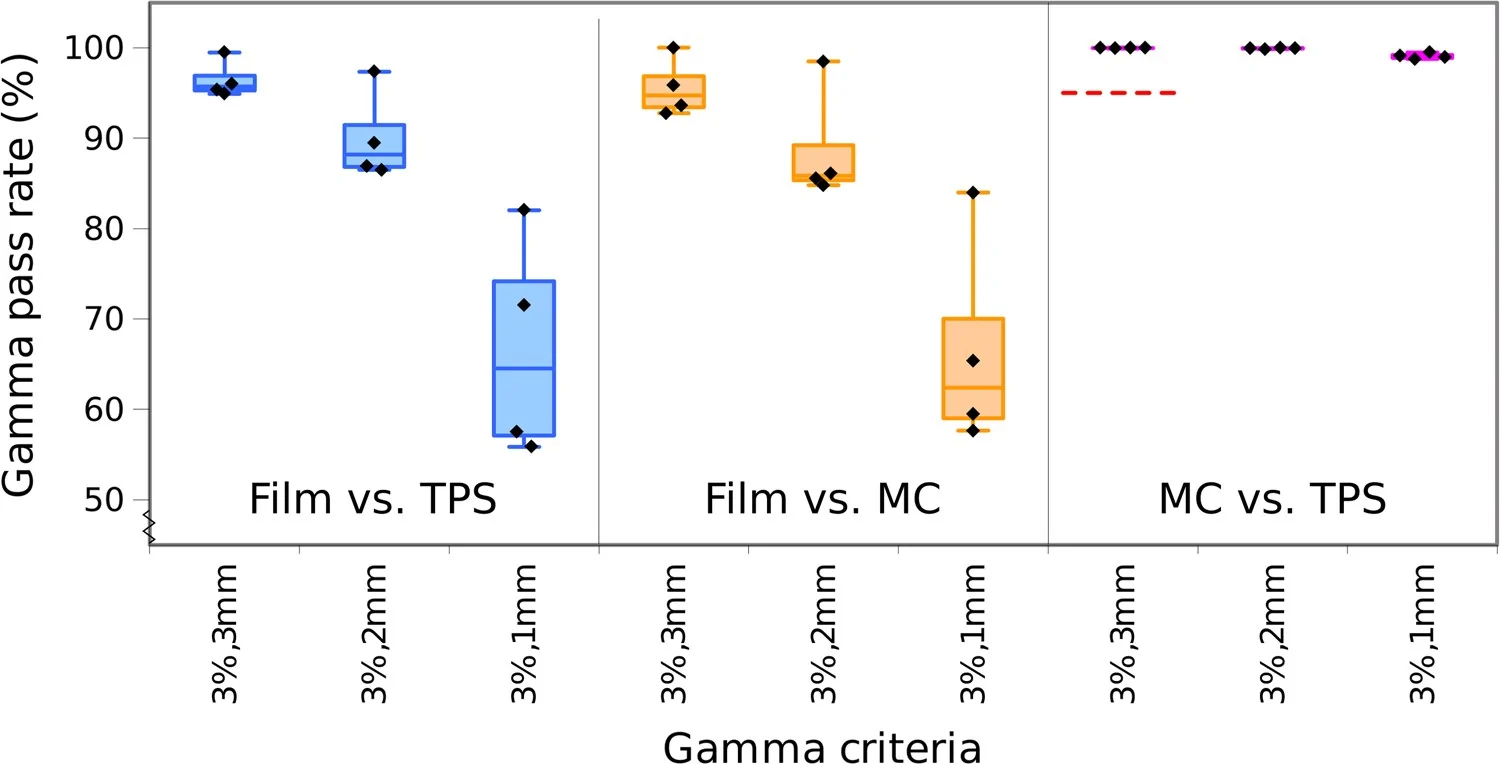
Summary of gamma analysis results for the end-to-end test deliveries to the CIRS phantom, comparing EBT4 film, the treatment planning system (TPS) dose calculation and the independent Monte-Carlo (MC) dose calculation. The box-plots show the median (center line), inter-quartile range (box) and range (whiskers).

Visually, the end-to-end tests’ film measurements (Figure 7A), TPS calculations (Figure 7C) and MC calculations (Figure 7F) were indistinguishable for the 10 vertex LRT plan. The gamma analyses showed subtle differences between film, TPS (Figure 7D) and MC (Figure 7G) for gamma criteria of 3%, 3 mm. 1D profiles in the posterior-anterior direction also showed good agreement between film, TPS and MC (Figure 7E and H). In summary, for both the 6 and 10 vertex plans good agreement between film measurements and both TPS and MC dose calculations was observed, with >94% of points having γ ≤ 1 at 3%, 3 mm for all films (Figure 8). Agreement between film and TPS or MC worsened as the distance-to-agreement criterion was tightened from 3 to 1 mm. Agreement between MC and TPS remained good at 3%, 1 mm.

## Discussion

### LRT planning implementation

For proton LRT we prescribed a mean dose of 20 Gy to the vertices. For VMAT LRT (planned as described by Ref.^20^) the prescription specified the near-maximum dose (*D*_0.5%_) to the GTV and was set to a value 20 Gy, although this could be relaxed to 16 Gy if necessary to meet the priority 1 objectives in Table 1. While the differing prescription metrics reflect variation in implementation at the Christie and Oxford, both approaches are consistent with prescriptions reported in other studies.^34–36^

### Dose to skin

Skin dose is an important consideration when delivering proton LRT as a single high-dose fraction. In this study, the skin $D_{0.5\;{\text{cm}}^{3}}^{}$ for proton LRT ranged from 3.0 to 9.3 Gy (mean 6.1 Gy), which was generally higher than for VMAT LRT but remained modest compared with the cumulative skin doses typically delivered during conventional fractionated radiotherapy. Since LRT is intended to be followed by a course of standard radiotherapy, the overall skin dose will depend on the degree of overlap between the LRT beam paths and those used in the subsequent course. Careful selection of field angles may therefore help limit cumulative skin dose. Historically, one of the motivations for SFRT was to reduce the skin toxicity associated with kilovoltage photon therapy.^37^ This suggests that proton LRT, where only small areas of skin receive high dose, may result in less toxicity than a uniform field of the same dose. Nevertheless, the biological response of skin to this delivery approach remains uncertain and should be monitored closely in any prospective clinical application.

### Dosimetric comparison: proton LRT vs VMAT LRT

For target volumes of less than approximately 2000 cm^3^ the proton and VMAT LRT approaches resulted in a similar number of vertices. For larger target volumes, proton LRT was capable of producing plans with an increased number of vertices. Whether the vertex density has a clinical impact is unknown. However, if proton LRT was limited to deliver the same number of vertices as VMAT LRT for large target volumes, it would allow more control over their placement eg, avoiding (or targeting) hypoxic volumes in the tumor.

### Deliverability

Our current clinical PSQA approach for standard proton plans is based on an independent MC dose calculation, alongside physical measurements using a 2D array and a PTW semiflex ionization chamber. For LRT plans, ionization chamber measurements were omitted since the small volume of the high-dose vertices would result in a large uncertainty in the chamber measurements arising from set-up accuracy. When using the PTW 1500XDR 2D array, we were aware that the volume-averaging over each 4.4 × 4.4 × 3 mm^3^ chamber and its relatively low spatial resolution (7.1 mm chamber pitch along diagonals) are not ideal for measurement of the small high-dose volumes in LRT plans. In addition, for PSQA of standard proton plans, we do not use the 2D array for verification of absolute dose for reasons noted by Aitkenhead et al.^27^ We therefore included film for its high spatial resolution and its ability to perform reference dosimetry (albeit with lower precision than a chamber).

From a structural point-of-view, proton LRT plans are little different to standard proton plans. Both consist of a set of energy layers, each of which is composed of a set of spots. To the delivery system, a set of LRT spots is no more complex than a standard IMPT field. Since there are fewer spots per energy layer in a lattice plan, the beam-on time is shorter than for standard IMPT. The structural similarity of the plans also means that use of our in-house independent MC dose calculation is equally valid for proton LRT as for standard IMPT. For clinical IMPT plans we use gamma criteria of 3%, 3 mm for evaluation of MC data. For proton LRT it may be prudent to evaluate using a tighter distance-to-agreement criterion, so we have reported 3, 2, and 1 mm here (Figure 6A).

In clinical practice, centers would need to decide on appropriate methods for their own PSQA of LRT fields. For VMAT LRT PSQA, Duriseti et al.^18^ used a combination of portal dosimetry, ionization chamber measurements and log-file evaluation. A different combination is required for proton LRT PSQA as portal dosimetry is not possible, and log-file analysis is not yet routinely available on Varian ProBeam systems. In this study, we chose a combination of MC, array and film measurements. MC calculations performed on a thoroughly commissioned system allow the dose calculation to be checked, leaving deliverability to be checked by other means. Film and array measurements have different features; 2D arrays allow for immediate evaluation of the result but have lower spatial resolution that is sub-optimal for LRT dosimetry, while film has excellent spatial resolution but is slow and determining the delivered dose to an accuracy of better than 5% is challenging.^38^ A combination of complementary approaches alongside a thorough machine QC programme is advisable to ensure the safe treatment of patients by proton LRT.

In the end-to-end test results (Figure 8), it was notable that gamma pass rates for film vs TPS or MC became worse as the distance-to-agreement criterion was tightened from 3 to 1 mm. This was likely due to the impact of small set-up errors. In particular, small rotational errors during phantom setup are difficult to avoid and will impact on gamma results for small distance-to-agreement criteria. In contrast, the MC vs TPS gamma results did not deteriorate by the same degree for small distance-to-agreement criteria since there is no set-up error involved in independent MC calculations.

### Evaluation of sites suitable for proton LRT

The retrospective audit of 536 treated at the Christie over a 24-month period only identified 5 potential patients for LRT. However, a large proportion of proton patients are pediatric (49.8% under 18 years old at the Christie) so do not form a representative population of the patient group likely to be candidates for SFRT, and the primary purpose of the audit was to identify cases for the study rather than to estimate the likely number of prospective patients suitable for LRT.

Proton therapy is not currently used in a palliative setting within the NHS since proton resources are limited (with 2 NHS high-energy facilities in operation) and photon therapy is considered a more efficient use of resources.^39^ If proton LRT was to be investigated for palliative treatments this would require some justification. However, a single fraction of proton LRT, combined with standard radiotherapy delivered by megavoltage photon therapy and/or immunotherapy, could be feasible from a resource point of view. For definitive treatments, proton LRT is likely to be most suitable for patients with very large tumors, such as sarcomas, where the tumor is likely to be radioresistant or in a re-irradiation setting.

## Conclusions

The study demonstrates that proton LRT is technically viable in the United Kingdom using currently available delivery systems. For all cases evaluated, proton and VMAT techniques both produced clinically acceptable LRT plans. Protons were able to reach the desired dose to vertices with a lower mean PTV dose, while the skin dose was generally higher compared to VMAT LRT. Large tumors such as sarcoma are most likely to be candidates for future clinical studies, but proton LRT is feasible for target volumes as small as 100 cm^3^.

## Supplementary Material

tqag125_Supplementary_Data

## Data Availability

The data underlying this article cannot be shared publicly due to GDPR and institutional data protection regulations concerning patient confidentiality. De-identified data may be available on reasonable request to the corresponding author, subject to approval by the relevant ethics committee and data-sharing agreements.
