# Supplementary material for: Proton pencil beam scanning lattice radiotherapy: technical implementation and comparison against photon-based delivery
Source: Br J Radiol. 2026 May 23;99(1185):1806–19. doi: 10.1093/bjr/tqag125 (PMC13489390; doi:10.1093/bjr/tqag125)
Supplement: Supplementary file 1 [file tqag125_supplementary_data.pdf]

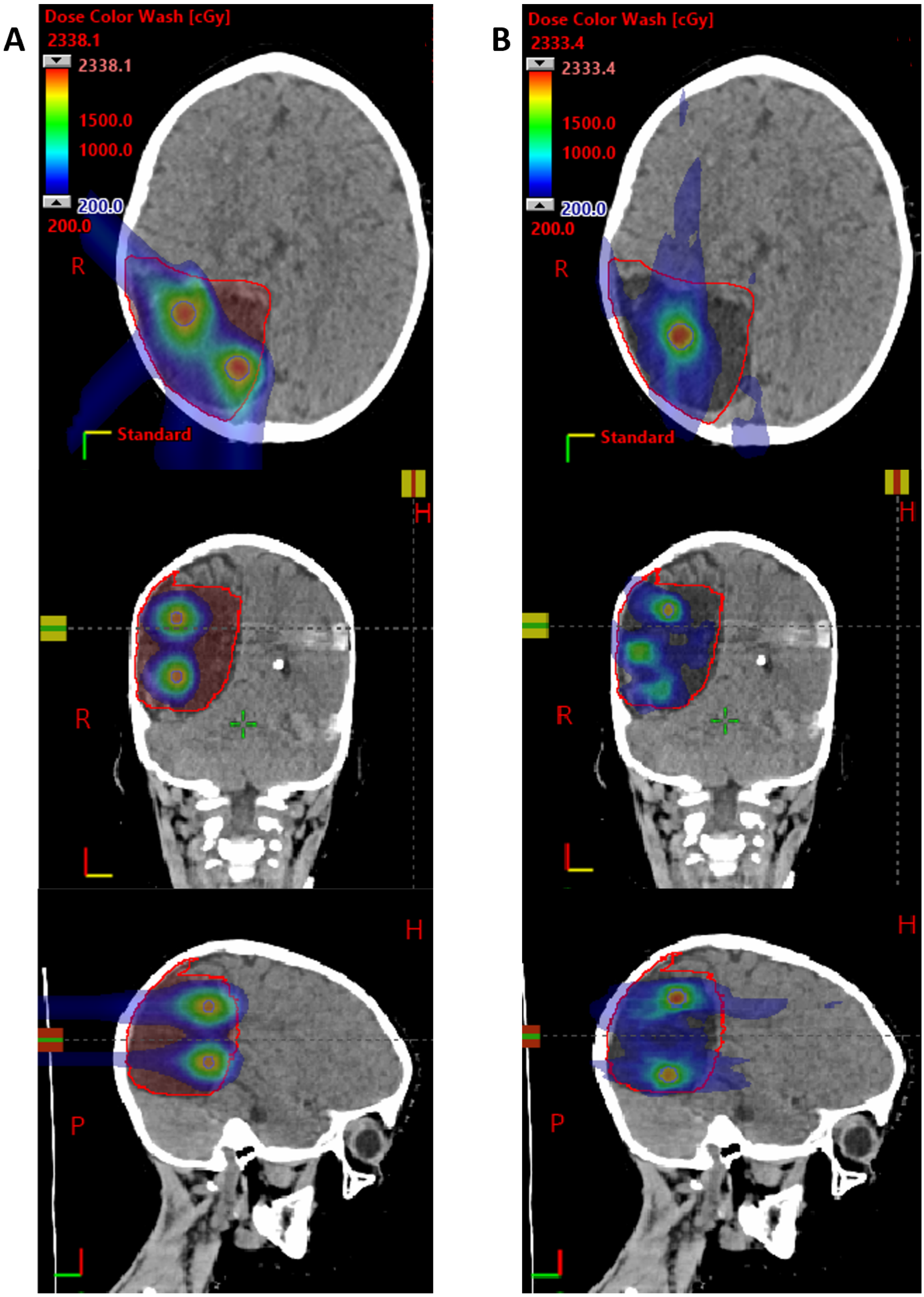

Figure A1 (supplemental): *The TPS dose distributions for a brain case (GTV = 130 cm^3) planned using (a) proton LRT and (b) VMAT LRT techniques. The plans contained 3 (for protons) and 4 (for VMAT) high-dose vertices.*
